# Supplementary material for: Transcriptome analysis of archived tumors by Visium, GeoMx DSP, and Chromium reveals patient heterogeneity
Source: Nat Commun. 2025 May 12;16:4400. doi: 10.1038/s41467-025-59005-9 (PMC12069714; doi:10.1038/s41467-025-59005-9)
Supplement: Supplementary file 1 — Supplementary Information [file 41467_2025_59005_MOESM1_ESM.pdf]

Fig S1

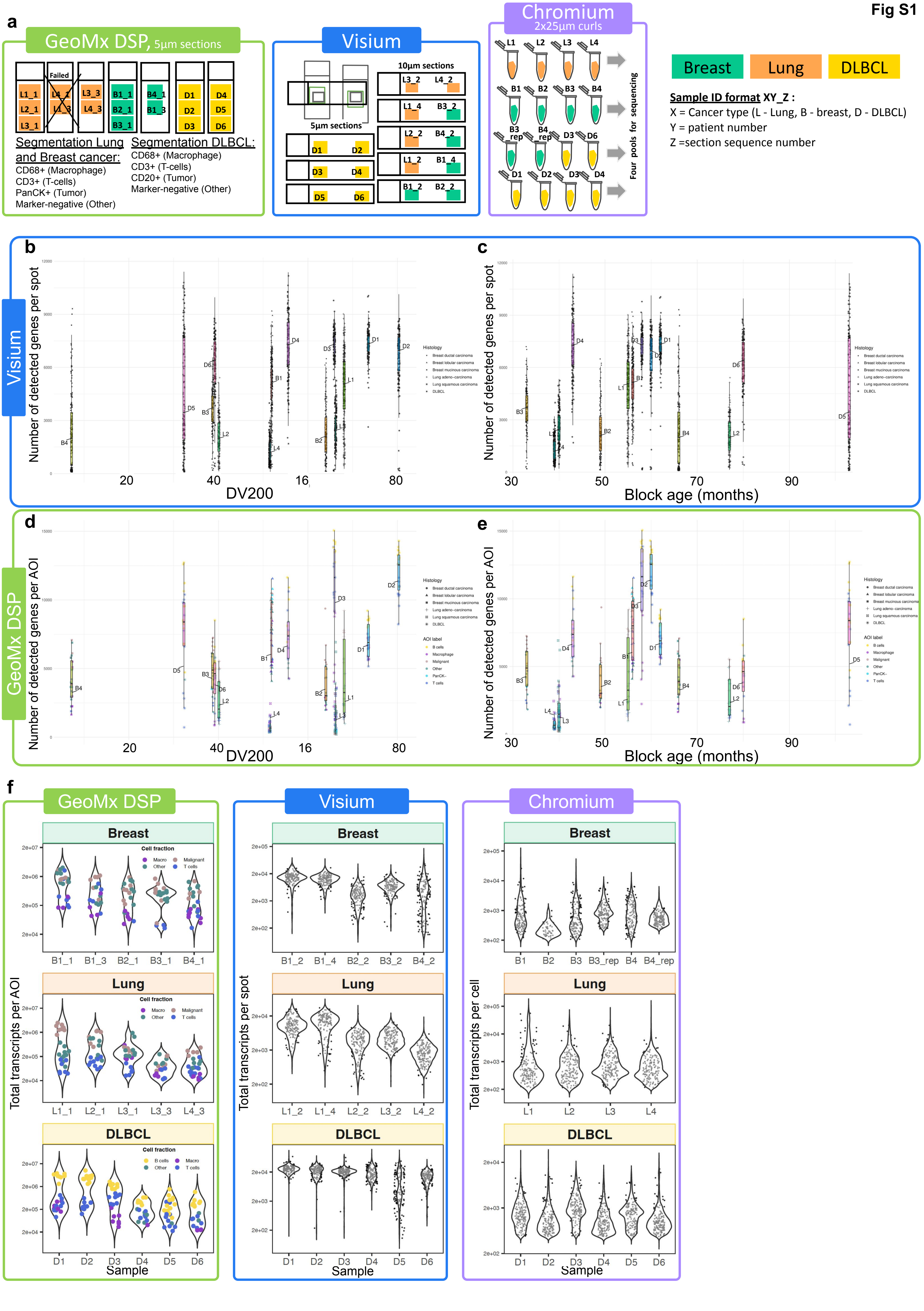

a
GeoMx DSP, 5µm sections
Failed
Segmentation Lung and Breast cancer:
CD68+ (Macrophage)
CD3+ (T-cells)
PanCK+ (Tumor)
Marker-negative (Other)
Segmentation DLBCL:
CD68+ (Macrophage)
CD3+ (T-cells)
CD20+ (Tumor)
Marker-negative (Other)
Visium
10µm sections
5µm sections
Chromium
2x25µm curls
Four pools for sequencing
Breast
Lung
DLBCL
Sample ID format XY_Z :
X = Cancer type (L - Lung, B - breast, D - DLBCL)
Y = patient number
Z =section sequence number
b
c
Visium
Number of detected genes per spot
DV200
Block age (months)
Histology
Breast ductal carcinoma
Breast lobular carcinoma
Breast mucinous carcinoma
Lung adeno−carcinoma
Lung squamous carcinoma
DLBCL
d
e
GeoMx DSP
Number of detected genes per AOI
AOI label
B cells
Macrophage
Malignant
Other
PanCK−
T cells
f
GeoMx DSP
Visium
Chromium
Breast
Lung
DLBCL
Cell fraction
Total transcripts per AOI
Total transcripts per spot
Total transcripts per cell
Sample

Fig S2

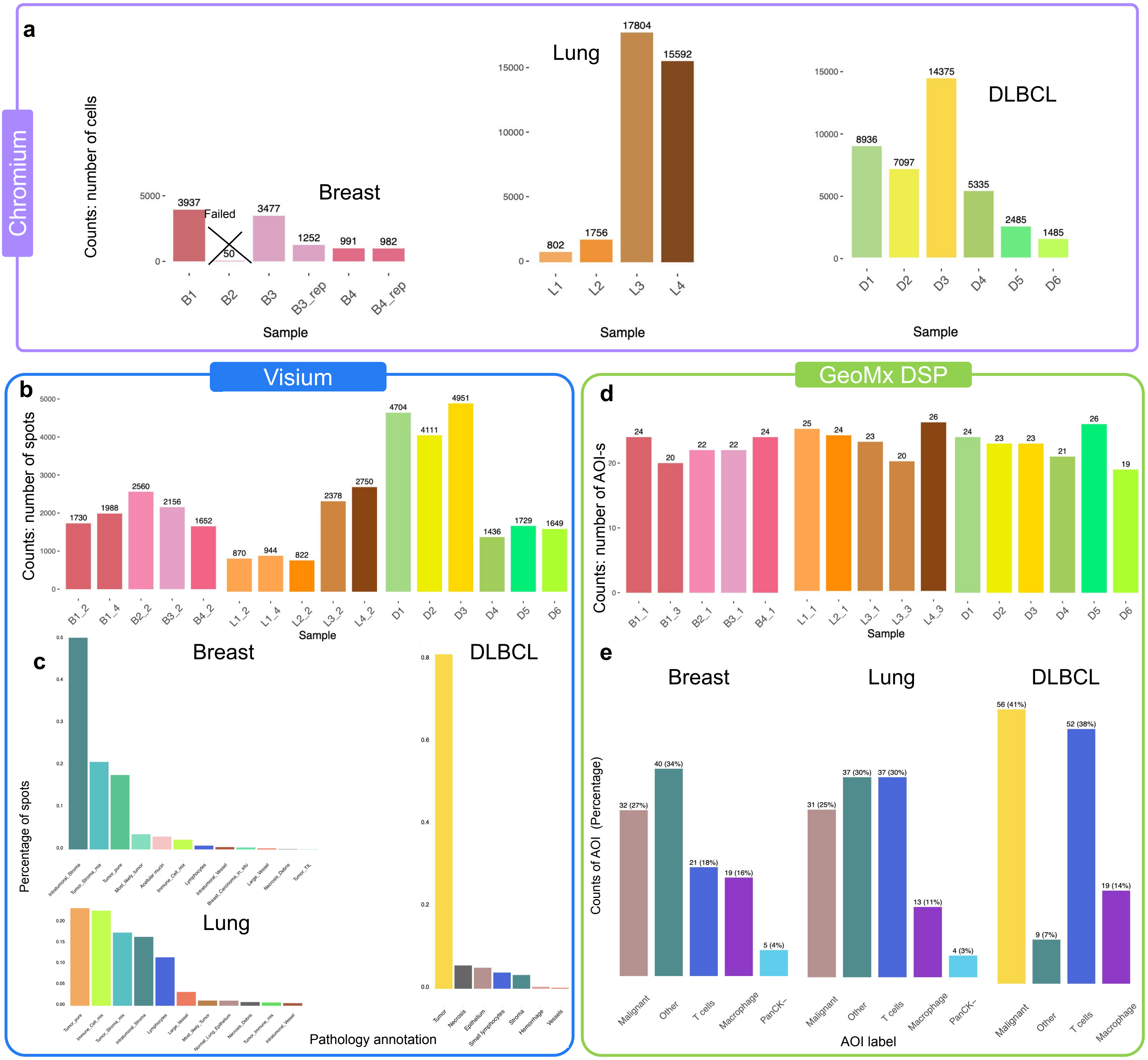

Chromium
a
Counts: number of cells
Breast
3937
Failed
50
3477
1252
991
982
B1
B2
B3
B3_rep
B4
B4_rep
Sample
Lung
802
1756
17804
15592
L1
L2
L3
L4
Sample
DLBCL
8936
7097
14375
5335
2485
1485
D1
D2
D3
D4
D5
D6
Sample
Visium
b
Counts: number of spots
1730
1988
2560
2156
1652
870
944
822
2378
2750
4704
4111
4951
1436
1729
1649
B1_2
B1_4
B2_2
B3_2
B4_2
L1_2
L1_4
L2_2
L3_2
L4_2
D1
D2
D3
D4
D5
D6
Sample
c
Breast
Percentage of spots
Lung
DLBCL
Tumor
Necrosis
Epithelium
Small lymphocytes
Stroma
Hemorrhage
Vessels
Pathology annotation
GeoMx DSP
d
Counts: number of AOI-s
24
20
22
22
24
25
24
23
20
26
24
23
23
21
26
19
B1_1
B1_3
B2_1
B3_1
B4_1
L1_1
L2_1
L3_1
L3_3
L4_3
D1
D2
D3
D4
D5
D6
Sample
e
Breast
Lung
DLBCL
Counts of AOI (Percentage)
32 (27%)
40 (34%)
21 (18%)
19 (16%)
5 (4%)
31 (25%)
37 (30%)
37 (30%)
13 (11%)
4 (3%)
56 (41%)
9 (7%)
52 (38%)
19 (14%)
Malignant
Other
T cells
Macrophage
PanCK-
AOI label

Fig S3

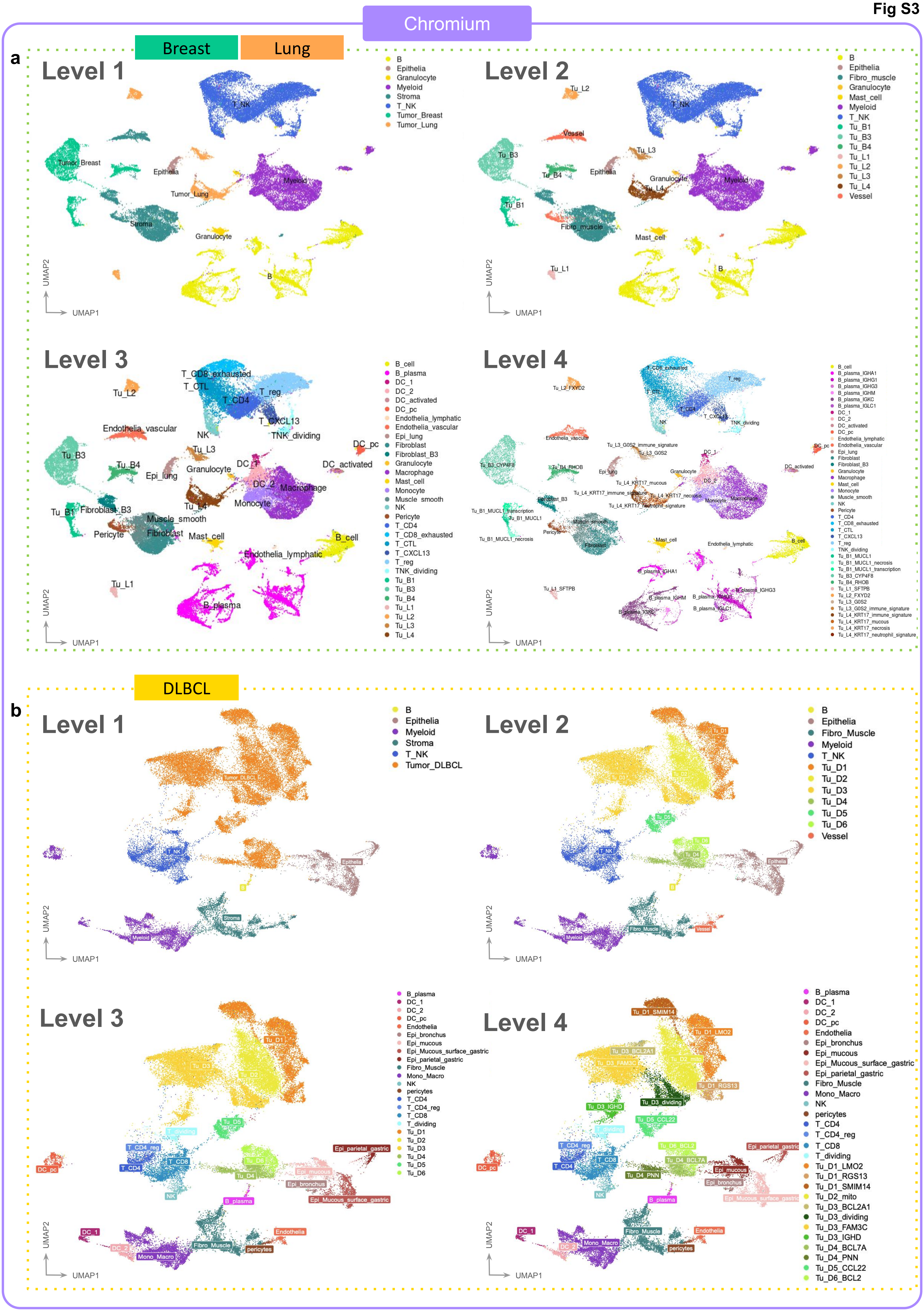

Chromium
a
Breast
Lung
Level 1
B
Epithelia
Granulocyte
Myeloid
Stroma
T_NK
Tumor_Breast
Tumor_Lung
Level 2
B
Epithelia
Fibro_muscle
Granulocyte
Mast_cell
Myeloid
T_NK
Tu_B1
Tu_B3
Tu_B4
Tu_L1
Tu_L2
Tu_L3
Tu_L4
Vessel
Level 3
B_cell
B_plasma
DC_1
DC_2
DC_activated
DC_pc
Endothelia_lymphatic
Endothelia_vascular
Epi_lung
Fibroblast
Fibroblast_B3
Granulocyte
Macrophage
Mast_cell
Monocyte
Muscle_smooth
NK
Pericyte
T_CD4
T_CD8_exhausted
T_CTL
T_CXCL13
T_reg
TNK_dividing
Tu_B1
Tu_B3
Tu_B4
Tu_L1
Tu_L2
Tu_L3
Tu_L4
Level 4
B_cell
B_plasma_IGHA1
B_plasma_IGHG1
B_plasma_IGHG3
B_plasma_IGHM
B_plasma_IGKC
B_plasma_IGLC1
DC_1
DC_2
DC_activated
DC_pc
Endothelia_lymphatic
Endothelia_vascular
Epi_lung
Fibroblast
Fibroblast_B3
Granulocyte
Macrophage
Mast_cell
Monocyte
Muscle_smooth
NK
Pericyte
T_CD4
T_CD8_exhausted
T_CTL
T_CXCL13
T_reg
TNK_dividing
Tu_B1_MUCL1
Tu_B1_MUCL1_necrosis
Tu_B1_MUCL1_transcription
Tu_B3_CYP4F8
Tu_B4_RHOB
Tu_L1_SFTPB
Tu_L2_FXYD2
Tu_L3_G0S2
Tu_L3_G0S2_immune_signature
Tu_L4_KRT17_immune_signature
Tu_L4_KRT17_mucous
Tu_L4_KRT17_necrosis
Tu_L4_KRT17_neutrophil_signature
UMAP1
UMAP2
b
DLBCL
Level 1
B
Epithelia
Myeloid
Stroma
T_NK
Tumor_DLBCL
Level 2
B
Epithelia
Fibro_Muscle
Myeloid
T_NK
Tu_D1
Tu_D2
Tu_D3
Tu_D4
Tu_D5
Tu_D6
Vessel
Level 3
B_plasma
DC_1
DC_2
DC_pc
Endothelia
Epi_bronchus
Epi_mucous
Epi_Mucous_surface_gastric
Epi_parietal_gastric
Fibro_Muscle
Mono_Macro
NK
pericytes
T_CD4
T_CD4_reg
T_CD8
T_dividing
Tu_D1
Tu_D2
Tu_D3
Tu_D4
Tu_D5
Tu_D6
Level 4
B_plasma
DC_1
DC_2
DC_pc
Endothelia
Epi_bronchus
Epi_mucous
Epi_Mucous_surface_gastric
Epi_parietal_gastric
Fibro_Muscle
Mono_Macro
NK
pericytes
T_CD4
T_CD4_reg
T_CD8
T_dividing
Tu_D1_LMO2
Tu_D1_RGS13
Tu_D1_SMIM14
Tu_D2_mito
Tu_D3_BCL2A1
Tu_D3_dividing
Tu_D3_FAM3C
Tu_D3_IGHD
Tu_D4_BCL7A
Tu_D4_PNN
Tu_D5_CCL22
Tu_D6_BCL2
UMAP1
UMAP2

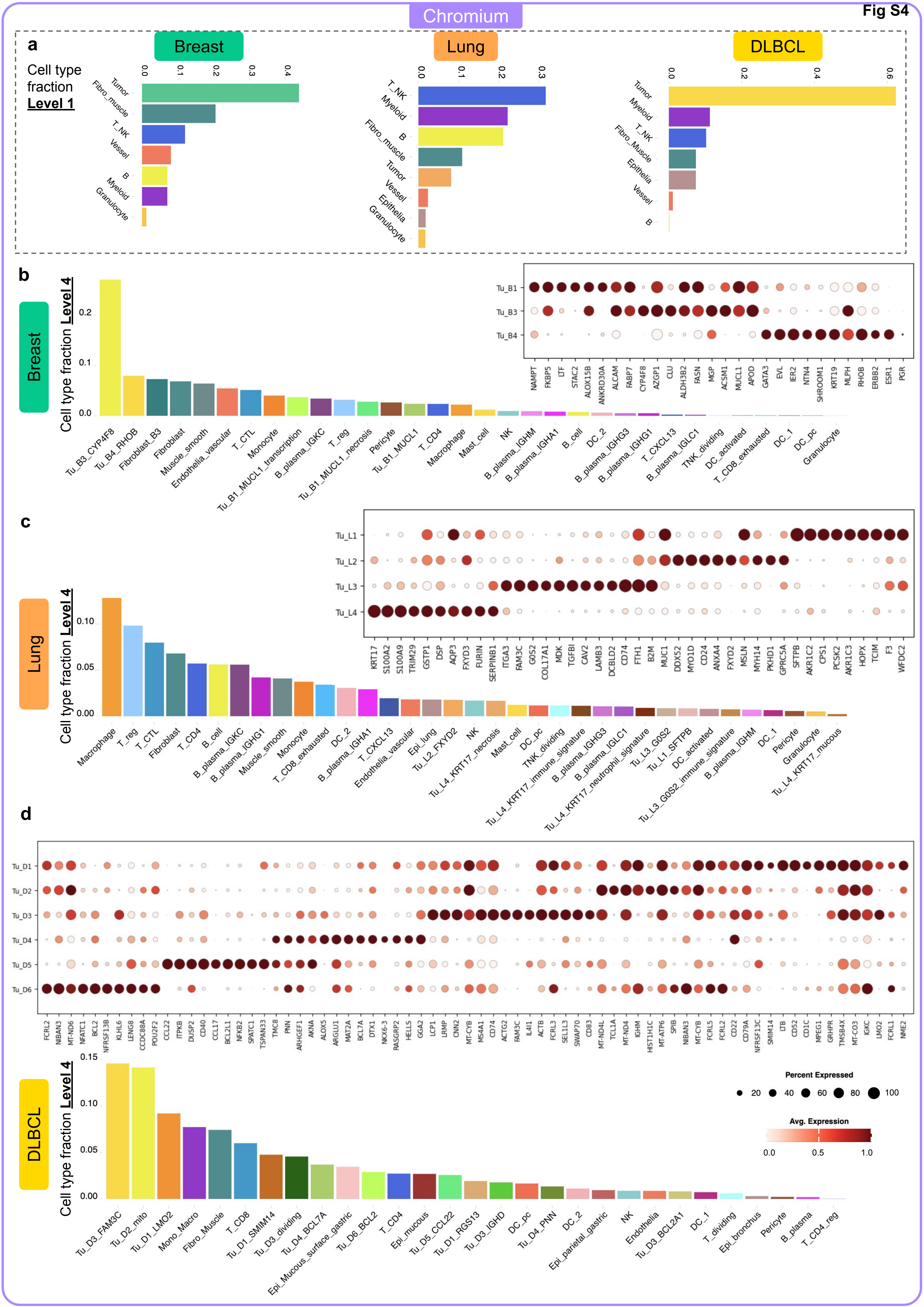
Chromium
a
Cell type fraction Level 1
Breast
Lung
DLBCL
b
Breast
Cell type fraction Level 4
c
Lung
Cell type fraction Level 4
d
DLBCL
Cell type fraction Level 4
Percent Expressed
20 40 60 80 100
Avg. Expression
0.0 0.5 1.0

Fig S5

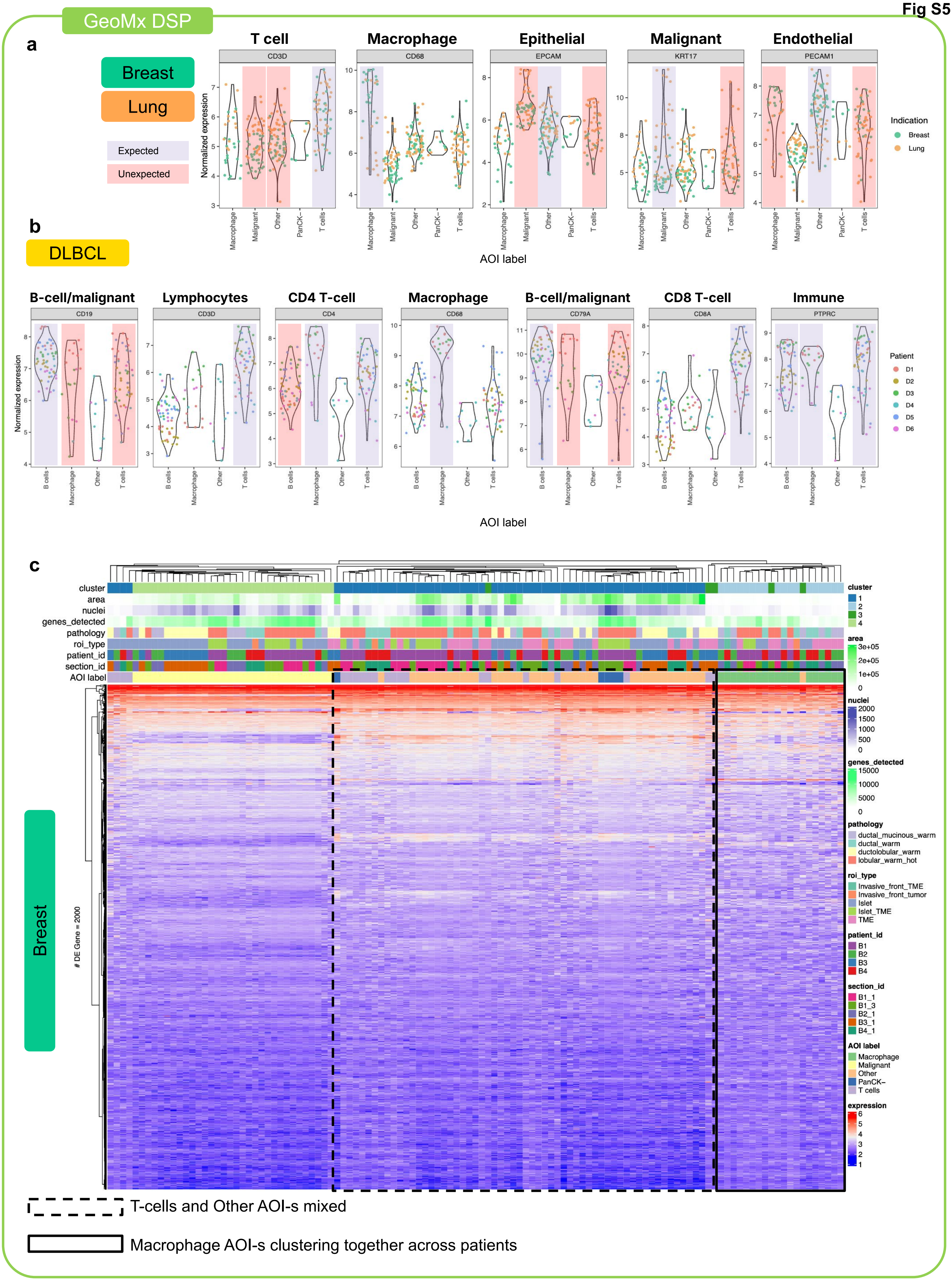
GeoMx DSP
a
Breast
Lung
Expected
Unexpected
T cell
CD3D
Macrophage
CD68
Epithelial
EPCAM
Malignant
KRT17
Endothelial
PECAM1
Normalized expression
Macrophage
Malignant
Other
PanCK−
T cells
AOI label
Indication
Breast
Lung
b
DLBCL
B-cell/malignant
CD19
Lymphocytes
CD3D
CD4 T-cell
CD4
Macrophage
CD68
B-cell/malignant
CD79A
CD8 T-cell
CD8A
Immune
PTPRC
B cells
Macrophage
Other
T cells
AOI label
Patient
D1
D2
D3
D4
D5
D6
c
cluster
area
nuclei
genes_detected
pathology
roi_type
patient_id
section_id
AOI label
Breast
DE Gene = 2000
cluster
1
2
3
4
area
3e+05
2e+05
1e+05
0
nuclei
2000
1500
1000
500
0
genes_detected
15000
10000
5000
0
pathology
ductal_mucinous_warm
ductal_warm
ductolobular_warm
lobular_warm_hot
roi_type
Invasive_front_TME
Invasive_front_tumor
Islet
Islet_TME
TME
patient_id
B1
B2
B3
B4
section_id
B1_1
B1_3
B2_1
B3_1
B4_1
AOI label
Macrophage
Malignant
Other
PanCK−
T cells
expression
6
5
4
3
2
1
T-cells and Other AOI-s mixed
Macrophage AOI-s clustering together across patients

Fig S6

Visium Registration

a

GeoMx	B1_1	B1_3	B2_1	B3_1	B4_1	L1_1	L2_1	L3_3	L4_3
Visium	B1_2	B1_4	B2_2	B3_2	B4_2	L1_2	L2_2	L3_2	L4_2

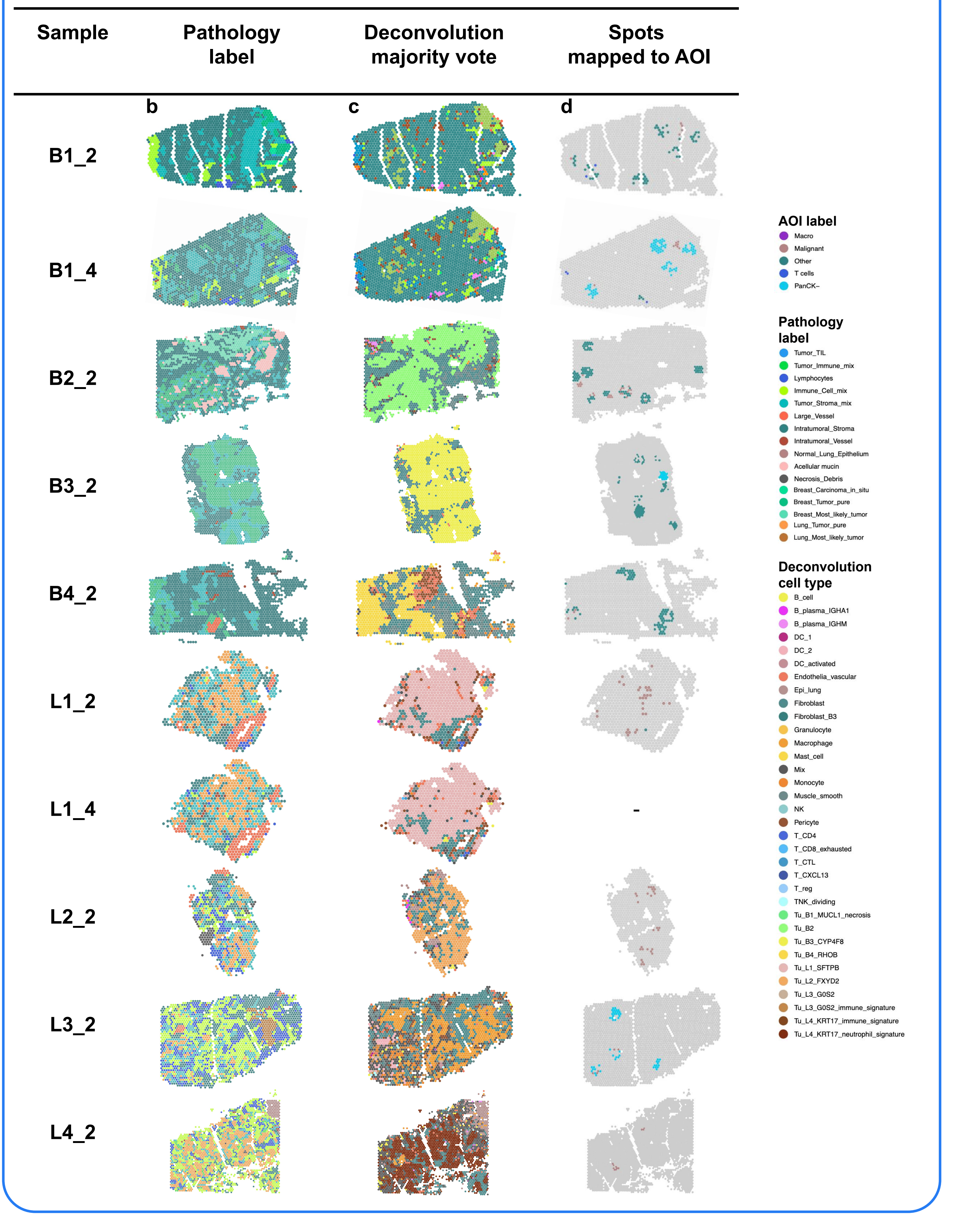

Fig S7

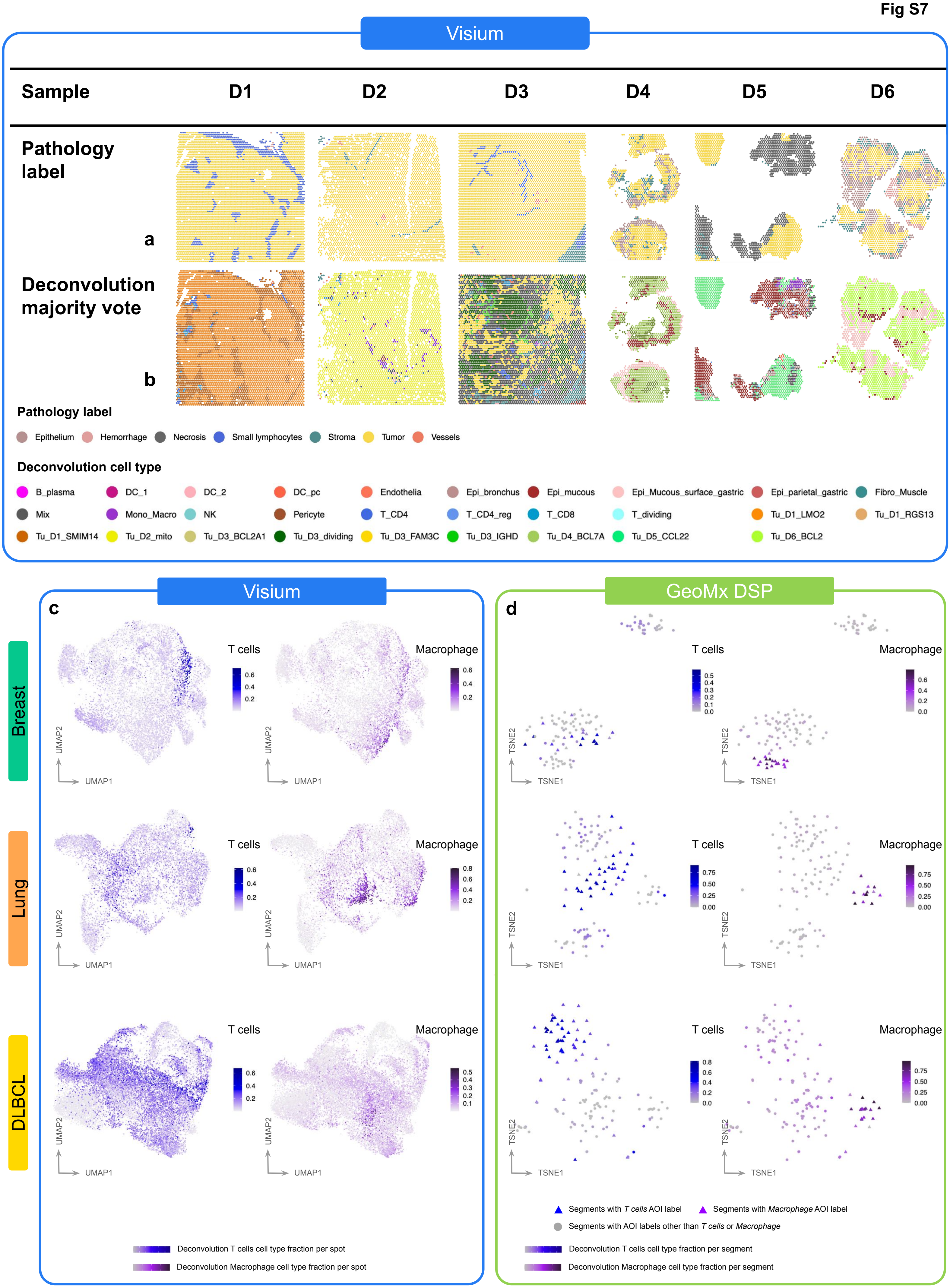
Visium
Sample
D1
D2
D3
D4
D5
D6
Pathology label
a
Deconvolution majority vote
b
Pathology label
Epithelium
Hemorrhage
Necrosis
Small lymphocytes
Stroma
Tumor
Vessels
Deconvolution cell type
B_plasma
DC_1
DC_2
DC_pc
Endothelia
Epi_bronchus
Epi_mucous
Epi_Mucous_surface_gastric
Epi_parietal_gastric
Fibro_Muscle
Mix
Mono_Macro
NK
Pericyte
T_CD4
T_CD4_reg
T_CD8
T_dividing
Tu_D1_LMO2
Tu_D1_RGS13
Tu_D1_SMIM14
Tu_D2_mito
Tu_D3_BCL2A1
Tu_D3_dividing
Tu_D3_FAM3C
Tu_D3_IGHD
Tu_D4_BCL7A
Tu_D5_CCL22
Tu_D6_BCL2
Visium
c
GeoMx DSP
d
Breast
Lung
DLBCL
T cells
Macrophage
UMAP1
UMAP2
TSNE1
TSNE2
Segments with T cells AOI label
Segments with Macrophage AOI label
Segments with AOI labels other than T cells or Macrophage
Deconvolution T cells cell type fraction per spot
Deconvolution Macrophage cell type fraction per spot
Deconvolution T cells cell type fraction per segment
Deconvolution Macrophage cell type fraction per segment

Fig S8

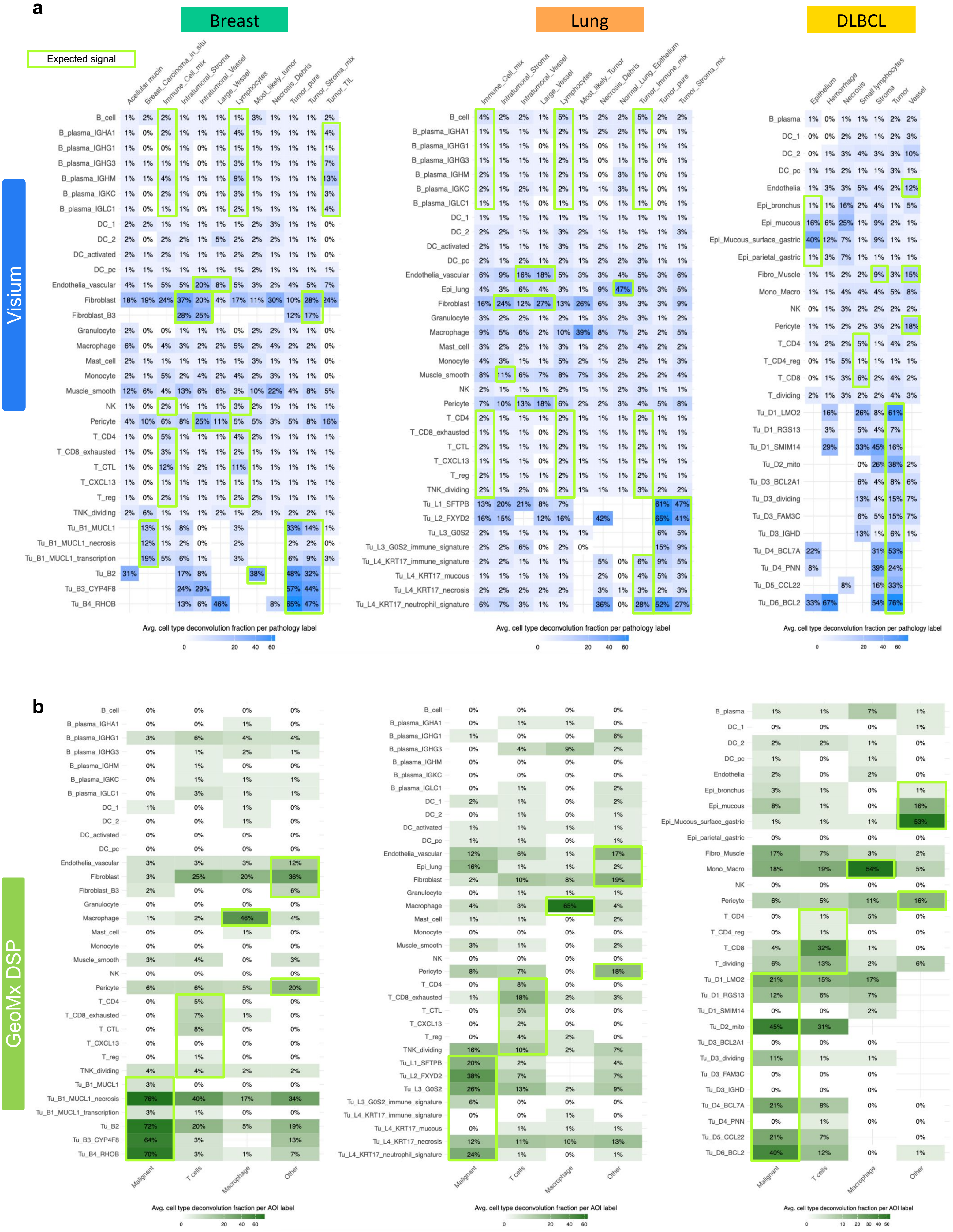
a
Breast
Lung
DLBCL
Expected signal
Visium
Avg. cell type deconvolution fraction per pathology label
b
GeoMx DSP
Malignant
T cells
Macrophage
Other
Avg. cell type deconvolution fraction per AOI label

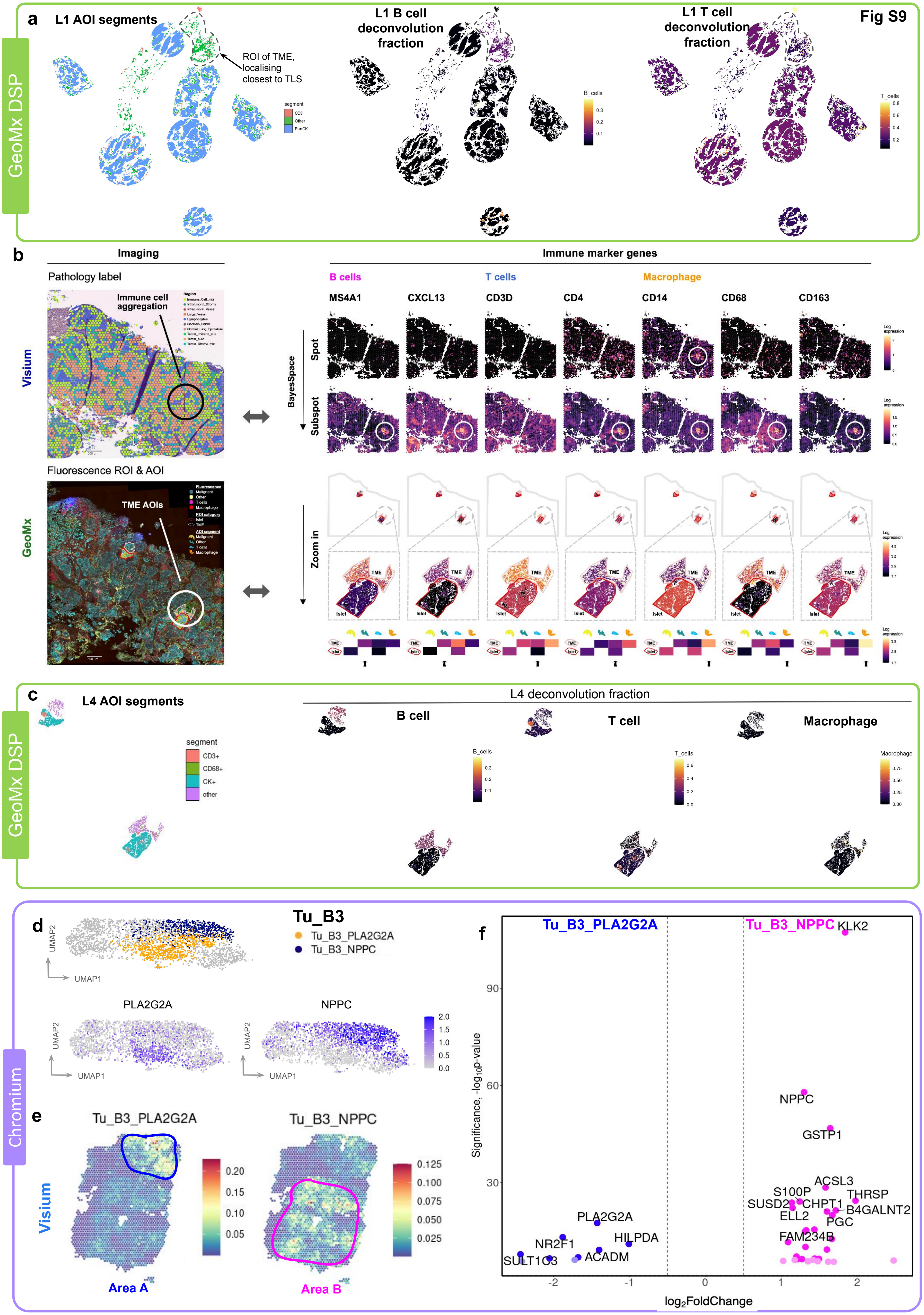

Fig S9
a
L1 AOI segments
ROI of TME, localising closest to TLS
L1 B cell deconvolution fraction
L1 T cell deconvolution fraction
GeoMx DSP
segment
CD3
Other
PanCK
B_cells
T_cells
b
Imaging
Pathology label
Immune cell aggregation
Visium
Fluorescence ROI & AOI
TME AOIs
GeoMx
Immune marker genes
B cells
T cells
Macrophage
MS4A1
CXCL13
CD3D
CD4
CD14
CD68
CD163
BayesSpace
Spot
Subspot
Zoom in
TME
Islet
Log expression
c
L4 AOI segments
segment
CD3+
CD68+
CK+
other
L4 deconvolution fraction
B cell
T cell
Macrophage
B_cells
T_cells
Macrophage
d
Tu_B3
Tu_B3_PLA2G2A
Tu_B3_NPPC
UMAP1
UMAP2
PLA2G2A
NPPC
Chromium
e
Tu_B3_PLA2G2A
Tu_B3_NPPC
Visium
Area A
Area B
f
Tu_B3_PLA2G2A
Tu_B3_NPPC
Significance, -log10p-value
log2FoldChange
KLK2
NPPC
GSTP1
ACSL3
S100P
THRSP
SUSD2
CHPT1
B4GALNT2
ELL2
PGC
FAM234B
PLA2G2A
HILPDA
NR2F1
ACADM
SULT1C3

Fig S10

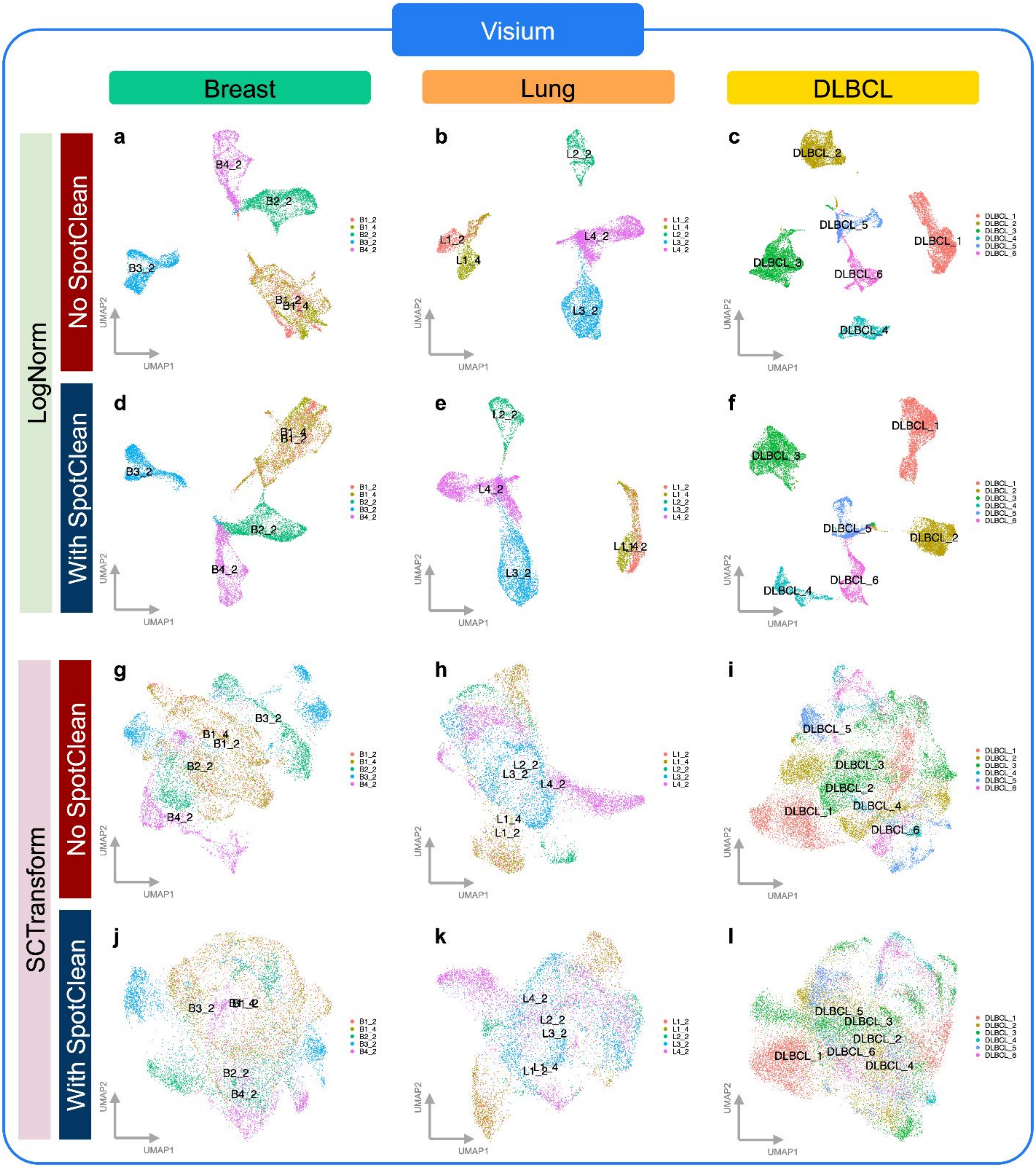

Visium
Breast
Lung
DLBCL
LogNorm
SCTransform
No SpotClean
With SpotClean
No SpotClean
With SpotClean
a
b
c
d
e
f
g
h
i
j
k
l
UMAP1
UMAP2
B1_2
B1_4
B2_2
B3_2
B4_2
L1_2
L1_4
L2_2
L3_2
L4_2
DLBCL_1
DLBCL_2
DLBCL_3
DLBCL_4
DLBCL_5
DLBCL_6

Fig S11

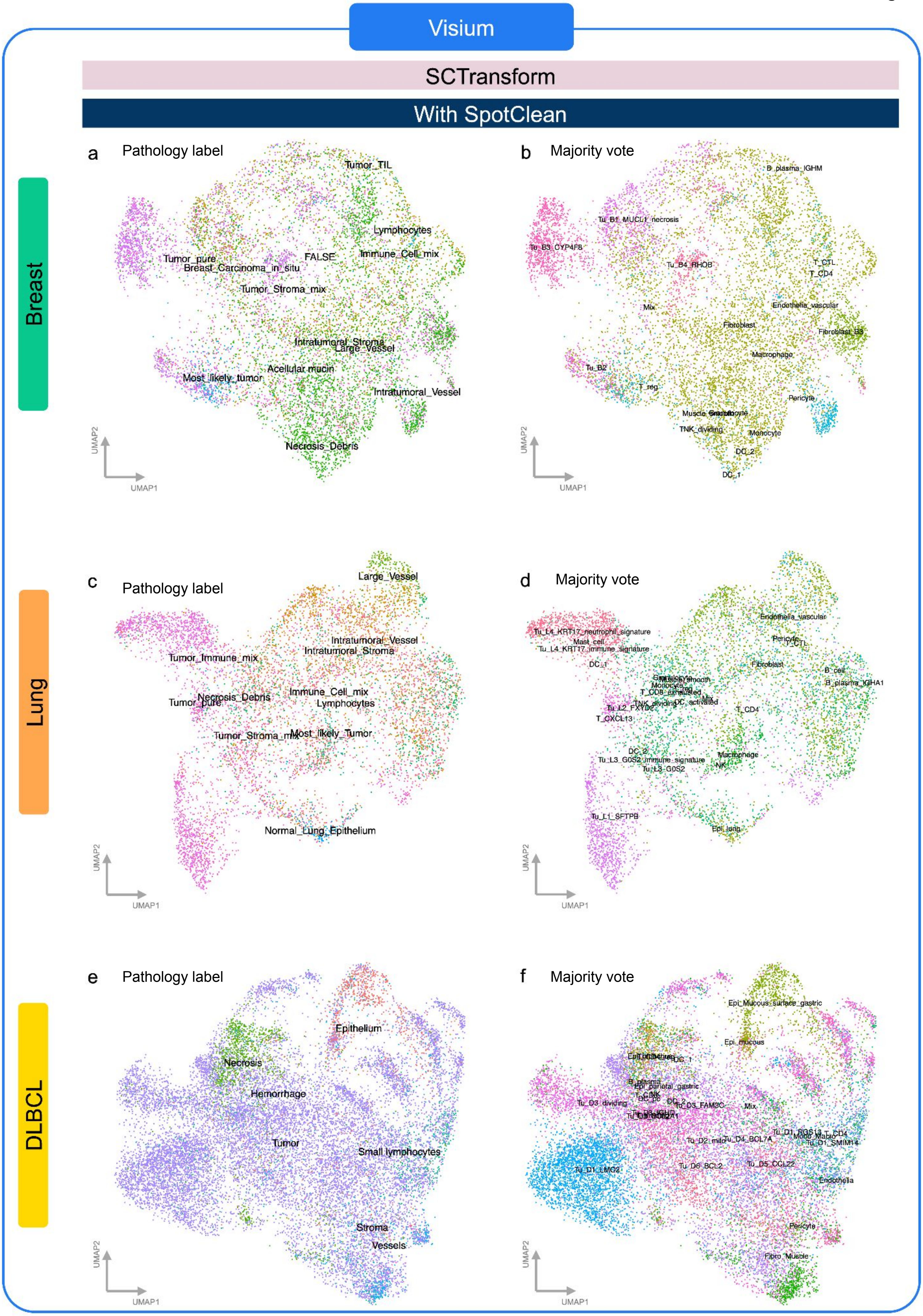

Visium
SCTransform
With SpotClean
Breast
Lung
DLBCL
a
Pathology label
Tumor_TIL
Lymphocytes
Immune_Cell_mix
FALSE
Tumor_pure
Breast_Carcinoma_in_situ
Tumor_Stroma_mix
Intratumoral_Stroma
Large_Vessel
Acellular mucin
Most_likely_tumor
Intratumoral_Vessel
Necrosis_Debris
UMAP2
UMAP1
b
Majority vote
B_plasma_IGHM
Tu_B1_MUCL1_necrosis
Tu_B3_CYP4F8
Tu_B4_RHOB
T_CTL
T_CD4
Mix
Endothelia_vascular
Fibroblast
Fibroblast_B3
Macrophage
Tu_B2
T_reg
Pericyte
TNK_dividing
Monocyte
DC_2
DC_1
c
Pathology label
Large_Vessel
Intratumoral_Vessel
Intratumoral_Stroma
Tumor_Immune_mix
Immune_Cell_mix
Lymphocytes
Necrosis_Debris
Tumor_pure
Most_likely_Tumor
Normal_Lung_Epithelium
d
Majority vote
Endothelia_vascular
Tu_L4_KRT17_neutrophil_signature
Mast_cell
Tu_L4_KRT17_immune_signature
DC_1
Fibroblast
B_cell
B_plasma_IGHA1
T_CD8_exhausted
TNK_dividing
Tu_L2_FXYD2
T_CXCL13
T_CD4
DC_2
Tu_L3_G0S2_immune_signature
Tu_L3_G0S2
Macrophage
NK
Tu_L1_SFTPB
Epi_lung
e
Pathology label
Epithelium
Necrosis
Hemorrhage
Tumor
Small lymphocytes
Stroma
Vessels
f
Majority vote
Epi_Mucous_surface_gastric
Epi_mucous
B_plasma
Epi_parietal_gastric
Tu_D3_dividing
Tu_D3_FAM3C
Mix
Tu_D2_mito
Tu_D4_BCL7A
Tu_D1_RGS13
T_CD4
Tu_D1_SMIM14
Tu_D1_LMO2
Tu_D6_BCL2
Tu_D5_CCL22
Endothelia
Pericyte
Fibro_Muscle

Fig S12

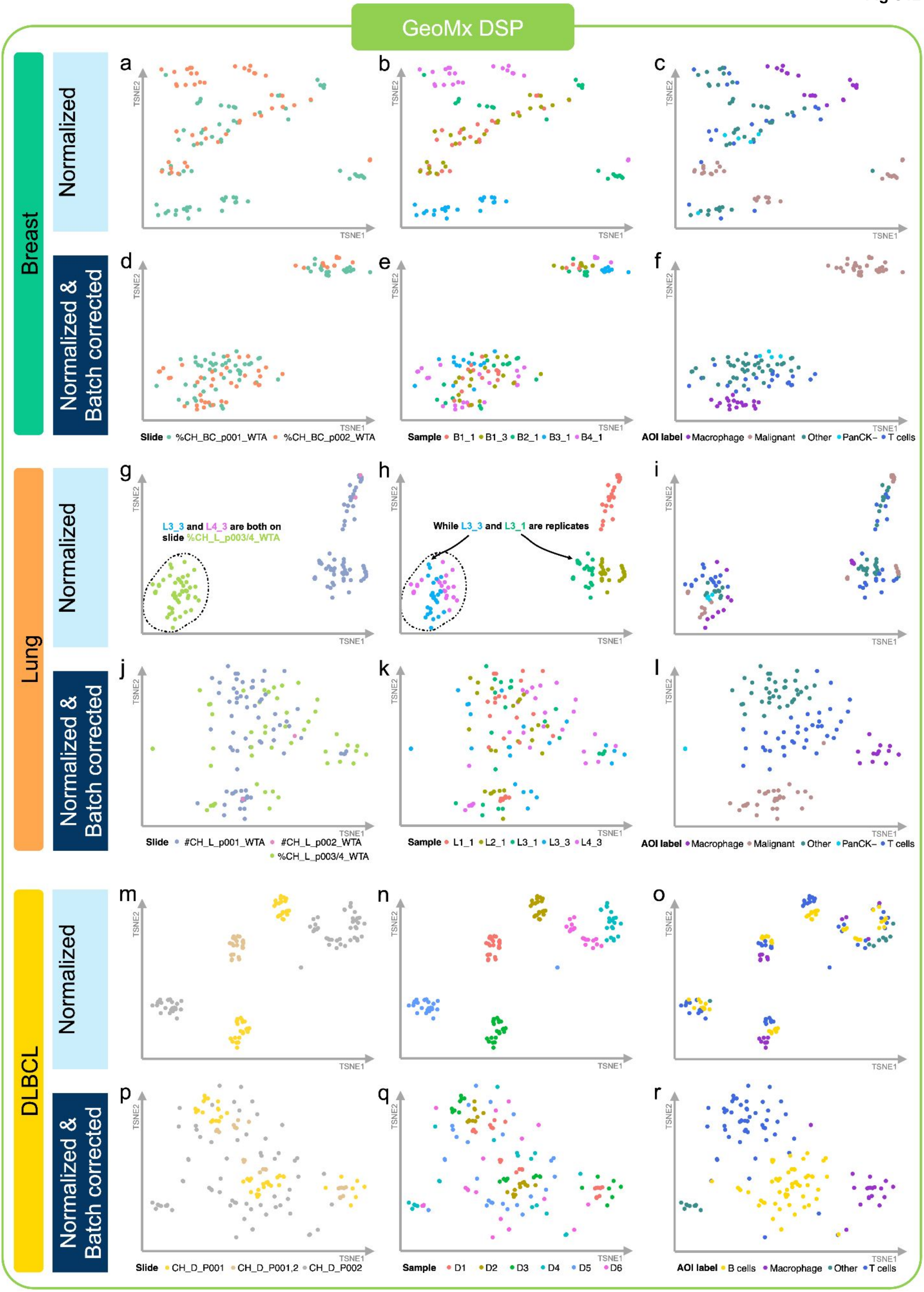

GeoMx DSP
Breast
Lung
DLBCL
Normalized
Normalized & Batch corrected
a
b
c
d
e
f
g
h
i
j
k
l
m
n
o
p
q
r
TSNE1
TSNE2
Slide • %CH_BC_p001_WTA • %CH_BC_p002_WTA
Sample • B1_1 • B1_3 • B2_1 • B3_1 • B4_1
AOI label • Macrophage • Malignant • Other • PanCK– • T cells
L3_3 and L4_3 are both on slide %CH_L_p003/4_WTA
While L3_3 and L3_1 are replicates
Slide • #CH_L_p001_WTA • #CH_L_p002_WTA • %CH_L_p003/4_WTA
Sample • L1_1 • L2_1 • L3_1 • L3_3 • L4_3
AOI label • Macrophage • Malignant • Other • PanCK– • T cells
Slide • CH_D_P001 • CH_D_P001,2 • CH_D_P002
Sample • D1 • D2 • D3 • D4 • D5 • D6
AOI label • B cells • Macrophage • Other • T cells

Fig S13

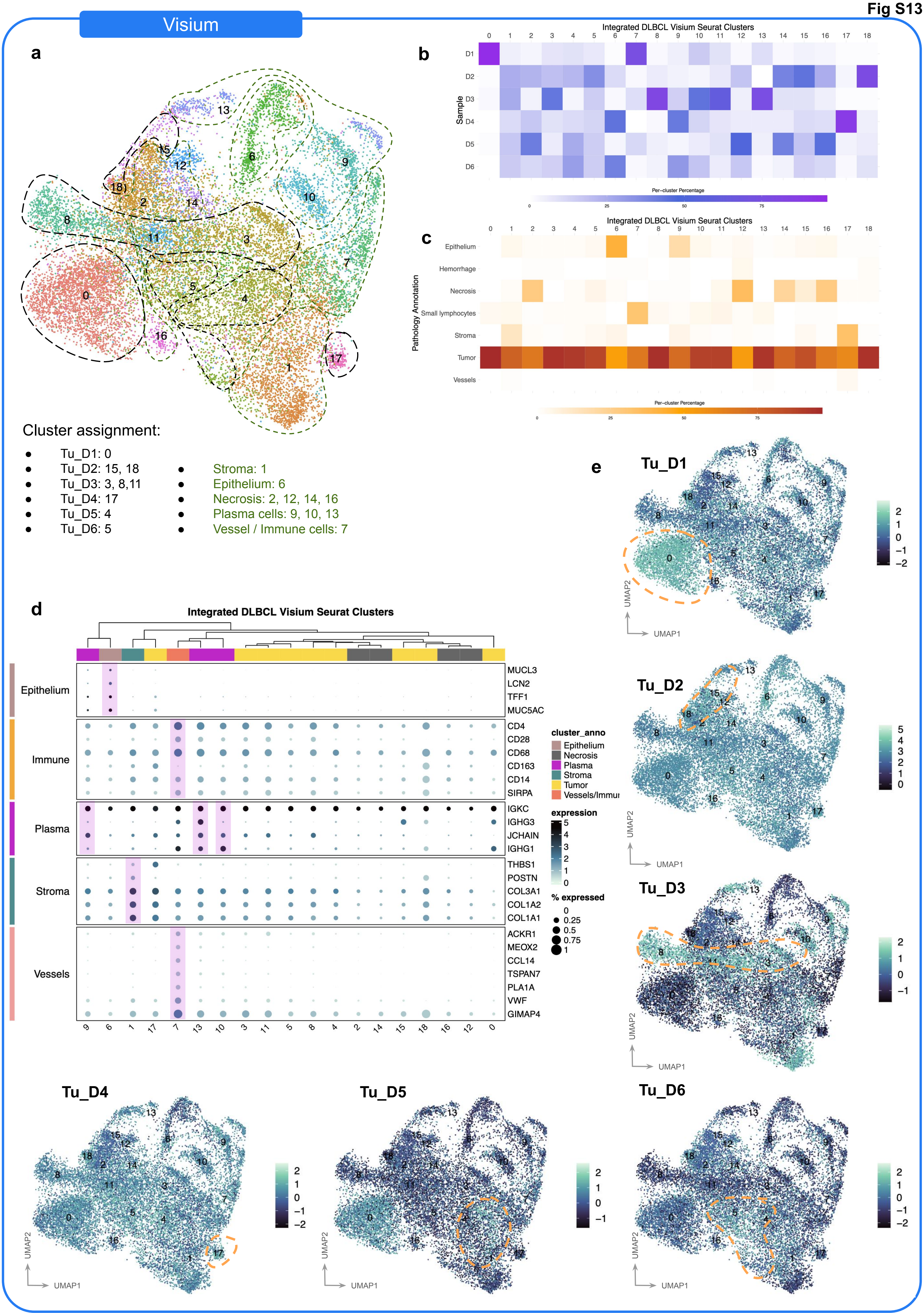
Visium
a
b
c
d
e
Integrated DLBCL Visium Seurat Clusters
Sample
D1
D2
D3
D4
D5
D6
Per-cluster Percentage
Pathology Annotation
Epithelium
Hemorrhage
Necrosis
Small lymphocytes
Stroma
Tumor
Vessels
Cluster assignment:
Tu_D1: 0
Tu_D2: 15, 18
Tu_D3: 3, 8,11
Tu_D4: 17
Tu_D5: 4
Tu_D6: 5
Stroma: 1
Epithelium: 6
Necrosis: 2, 12, 14, 16
Plasma cells: 9, 10, 13
Vessel / Immune cells: 7
Epithelium
Immune
Plasma
Stroma
Vessels
MUCL3
LCN2
TFF1
MUC5AC
CD4
CD28
CD68
CD163
CD14
SIRPA
IGKC
IGHG3
JCHAIN
IGHG1
THBS1
POSTN
COL3A1
COL1A2
COL1A1
ACKR1
MEOX2
CCL14
TSPAN7
PLA1A
VWF
GIMAP4
cluster_anno
Epithelium
Necrosis
Plasma
Stroma
Tumor
Vessels/Immu
expression
% expressed
Tu_D1
Tu_D2
Tu_D3
Tu_D4
Tu_D5
Tu_D6
UMAP1
UMAP2

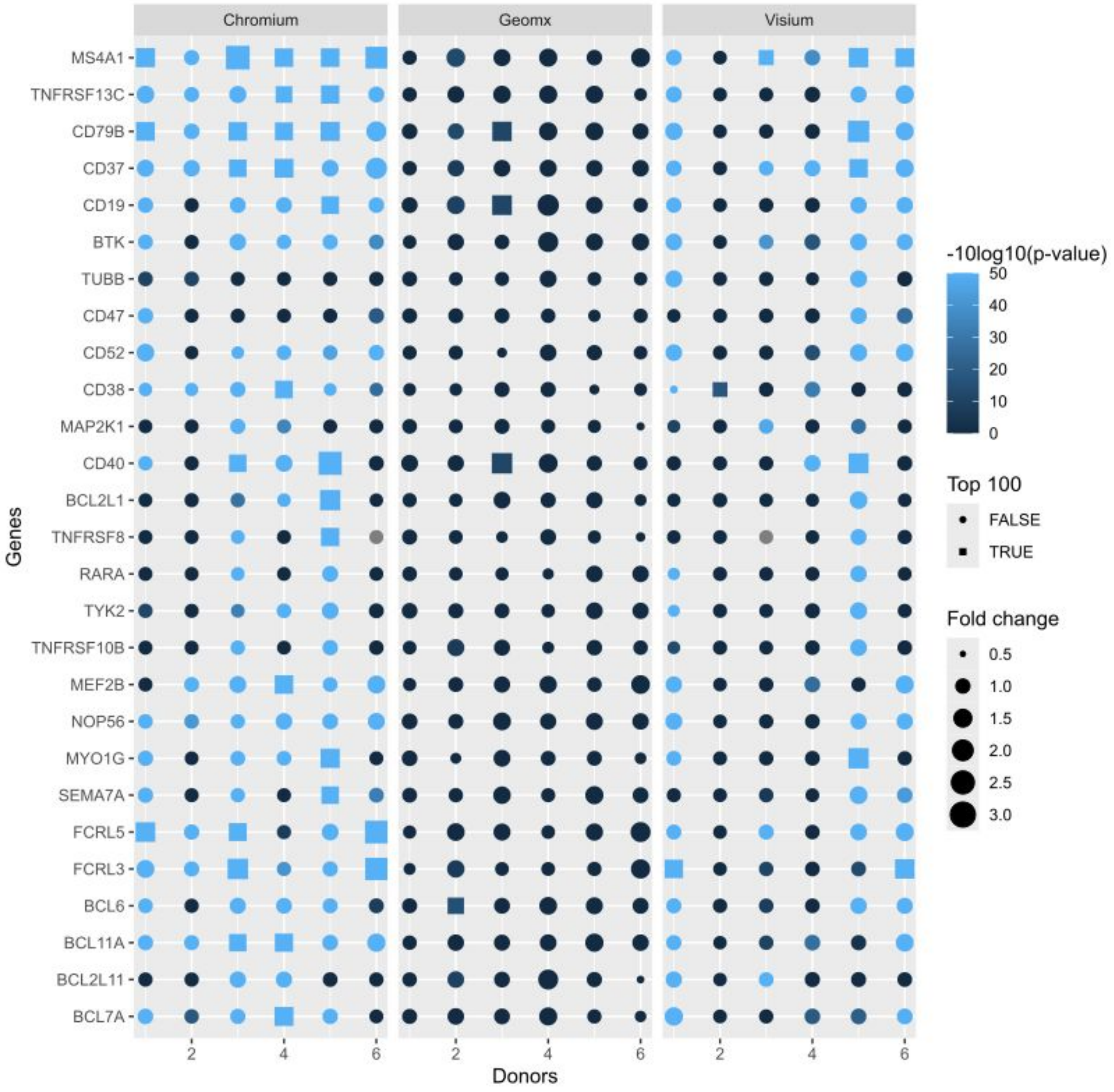
Chromium
Geomx
Visium
MS4A1
TNFRSF13C
CD79B
CD37
CD19
BTK
TUBB
CD47
CD52
CD38
MAP2K1
CD40
BCL2L1
TNFRSF8
RARA
TYK2
TNFRSF10B
MEF2B
NOP56
MYO1G
SEMA7A
FCRL5
FCRL3
BCL6
BCL11A
BCL2L11
BCL7A
Genes
2
4
6
Donors
-10log10(p-value)
50
40
30
20
10
0
Top 100
FALSE
TRUE
Fold change
0.5
1.0
1.5
2.0
2.5
3.0

Fig S15

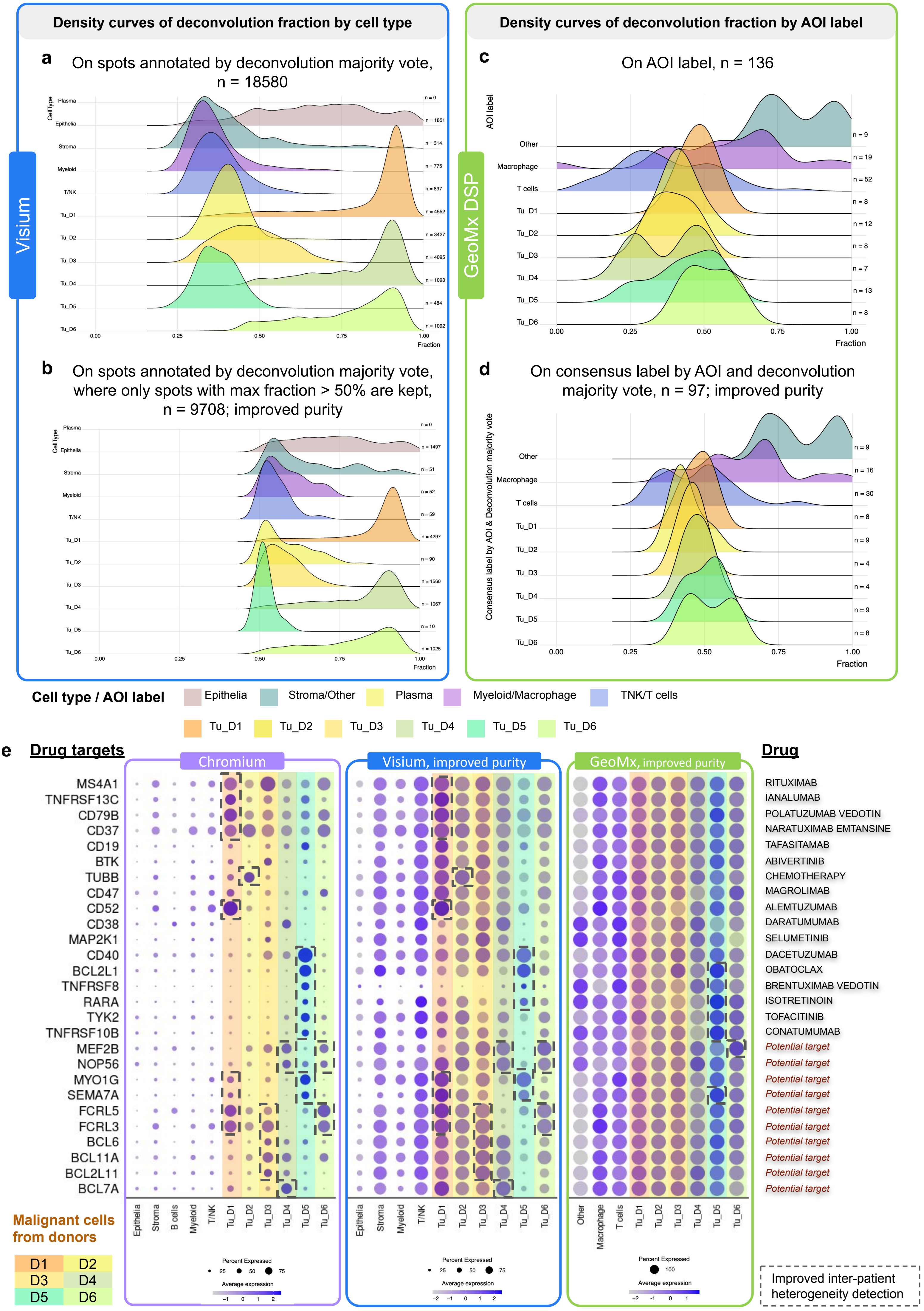
Density curves of deconvolution fraction by cell type
Visium
a
On spots annotated by deconvolution majority vote, n = 18580
CellType
Plasma
Epithelia
Stroma
Myeloid
T/NK
Tu_D1
Tu_D2
Tu_D3
Tu_D4
Tu_D5
Tu_D6
n = 0
n = 1851
n = 314
n = 775
n = 897
n = 4552
n = 3427
n = 4095
n = 1093
n = 484
n = 1092
0.00
0.25
0.50
0.75
1.00
Fraction
b
On spots annotated by deconvolution majority vote, where only spots with max fraction > 50% are kept, n = 9708; improved purity
n = 0
n = 1497
n = 51
n = 52
n = 59
n = 4297
n = 90
n = 1560
n = 1067
n = 10
n = 1025
Density curves of deconvolution fraction by AOI label
GeoMx DSP
c
On AOI label, n = 136
AOI label
Other
Macrophage
T cells
Tu_D1
Tu_D2
Tu_D3
Tu_D4
Tu_D5
Tu_D6
n = 9
n = 19
n = 52
n = 8
n = 12
n = 8
n = 7
n = 13
n = 8
d
On consensus label by AOI and deconvolution majority vote, n = 97; improved purity
Consensus label by AOI & Deconvolution majority vote
n = 9
n = 16
n = 30
n = 8
n = 9
n = 4
n = 4
n = 9
n = 8
Cell type / AOI label
Epithelia
Stroma/Other
Plasma
Myeloid/Macrophage
TNK/T cells
Tu_D1
Tu_D2
Tu_D3
Tu_D4
Tu_D5
Tu_D6
e
Drug targets
Chromium
Visium, improved purity
GeoMx, improved purity
Drug
MS4A1 RITUXIMAB
TNFRSF13C IANALUMAB
CD79B POLATUZUMAB VEDOTIN
CD37 NARATUXIMAB EMTANSINE
CD19 TAFASITAMAB
BTK ABIVERTINIB
TUBB CHEMOTHERAPY
CD47 MAGROLIMAB
CD52 ALEMTUZUMAB
CD38 DARATUMUMAB
MAP2K1 SELUMETINIB
CD40 DACETUZUMAB
BCL2L1 OBATOCLAX
TNFRSF8 BRENTUXIMAB VEDOTIN
RARA ISOTRETINOIN
TYK2 TOFACITINIB
TNFRSF10B CONATUMUMAB
MEF2B Potential target
NOP56 Potential target
MYO1G Potential target
SEMA7A Potential target
FCRL5 Potential target
FCRL3 Potential target
BCL6 Potential target
BCL11A Potential target
BCL2L11 Potential target
BCL7A Potential target
Epithelia
Stroma
B cells
Myeloid
T/NK
Tu_D1
Tu_D2
Tu_D3
Tu_D4
Tu_D5
Tu_D6
Other
Macrophage
T cells
Percent Expressed
25
50
75
100
Average expression
−2
−1
0
1
2
Malignant cells from donors
D1
D2
D3
D4
D5
D6
Improved inter-patient heterogeneity detection
