# Supplementary material for: Transcriptome analysis of archived tumors by Visium, GeoMx DSP, and Chromium reveals patient heterogeneity
Source: Nat Commun. 2025 May 12;16:4400. doi: 10.1038/s41467-025-59005-9 (PMC12069714; doi:10.1038/s41467-025-59005-9)
Supplement: Supplementary file 3 — Description of Additional Supplementary Files [file 41467_2025_59005_MOESM3_ESM.pdf]

Description of Additional Supplementary Files

File Name: Supplementary Figure 1

Description: Overview of data. (a) Schematic for GeoMx and Visium spatial transcriptomics and Chromium scRNA-seq data generation for breast, lung and DLBCL cancer types. Number of detected genes shown per spots in Visium (b, c) and AOI-s in GeoMx (d, e) shown in boxplots grouped by donor for all blocks with relation to the DV200 value (b, d) and block age in months (c, e). Dot shapes show histology of the sample (b-e) and color of the AOI label in GeoMx (d, e). (f) Total number of transcripts per AOI, spot, and cell in GeoMx, Visium, and Chromium, respectively, across all samples and indications. Source data are provided as a Source Data file.

File Name: Supplementary Figure 2

Description: Distribution of data points across donors, regions or AOI label type. Sample size with number of nuclei, spots and AOI-s in Chromium, Visium and GeoMx, respectively, for all indications per sample (a, b, d). (c) Percentage of spots in Visium falling into each pathology annotation for all indications. (e) Number and percentage of AOI-s in GeoMx falling into each curated segment as AOI label in all indications. AOI - area of illumination. DLBCL - Diffuse Large B-cell Lymphoma. DSP - digital spatial profiler. Source data are provided as a Source Data file.

File Name: Supplementary Figure 3

Description: Chromium scRNA-seq data annotated into different levels of cell type groups. Breast and Lung (a) and DLBCL (b) samples received level 4 annotation based on unsupervised clustering and canonical marker expression. The higher levels were derived by combining cell types of the lower level into biologically meaningful groups. Breast & Lung: 10 samples/46,643 cells; DLBCL: 6 samples/39,713 cells. Source data are provided as a Source Data file.

File Name: Supplementary Figure 4

Description: Cell type frequencies and malignant marker genes on level 1 and level 4 annotated Chromium. (a) Ordered by category percentage, the cell type frequency in each indication at Level 1 annotation. Ordered by category percentage, the marker genes for malignant nuclei in each donor, and cell type frequency at Level 4, ordered by percentage for breast (b), lung (c) and DLBCL (d). Source data are provided as a Source Data file.

File Name: Supplementary Figure 5

Description: Cell type mixtures in GeoMx segments by marker gene expression and unsupervised clustering. Cell type specific genes' expression in AOI-s grouped by collected AOI label in breast and lung (a) samples and in DLBCL (b). Known marker genes' expression specific for the selected segment is highlighted in light purple as "Expected" signal. "Unexpected" signal shows expression of the known marker in other segments. (c) Heatmap of normalized and batch corrected gene expression matrix on top 2000 differentially expressed genes between AOI label types, annotated by unsupervised clustering and metadata from Lab Worksheet of GeoMx breast samples (Supplementary Data 4b). Highlighted areas corresponding to AOI labels. Source data are provided as a Source Data file.

File Name: Supplementary Figure 6

Description: A gallery of breast and lung Visium samples with pathology annotation, deconvolution majority vote, and GeoMx matching. (a) Sample names matched between GeoMx and Visium for registration analysis as consecutive sections from the same block in breast and lung. Breast and lung Visium samples colored by pathology annotation (b), deconvolution majority vote (c), or mapped AOI label (d) for samples used for registration. Number of spots per sample are detailed in Supplementary Figure S2. Source data are provided as a Source Data file.

File Name: Supplementary Figure 7

Description: A gallery of DLBCL Visium samples with pathology annotation and deconvolution majority vote, and immune cell signals on reduced dimensions of integrated Visium and GeoMx. DLBCL Visium samples colored by pathology annotation label (a) and Level 4 deconvolution majority vote (b). Number of spots per sample are detailed in Supplementary Figure S2. UMAP plots (c) in Visium and TSNE plots (d) in GeoMx integrated between all donors for each indication, showing enrichment of T-cell and Macrophage cell type groups by deconvolution fractions. *T cells* and *Macrophage* AOI labels on GeoMx are shown by triangles. Different deconvolution fractions of tumor and fibroblast cell types between Area A and B. Visium - Breast: 5 samples/10,086 spots; Lung: 5 samples/7,764 spots; DLBCL: 6 samples/18,580 spots. GeoMx - Breast: 5 samples/112 AOIs; Lung: 5 samples/118 AOIs; DLBCL: 6 samples/136 AOIs. Source data are provided as a Source Data file.

File Name: Supplementary Figure 8

Description: Comparison of pathology annotation and deconvolution majority vote agreement in each indication. Agreement between pathology annotation or AOI label and cell type deconvolution results in Visium (a) and GeoMx (b), respectively, displayed as average cell type deconvolution fraction per pathology label or AOI label, colored by square root of the number, for breast, lung, and DLBCL samples, separately. Expected signal is surrounded by a green box. Source data are provided as a Source Data file.

File Name: Supplementary Figure 9

Description: Exploration of increased resolution with Visium, GeoMx and Chromium. (a) GeoMx ROI-s from L1 sample colored by segment type, B- or T-cell deconvolution fraction. Dotted line around the ROI of TME localising at TLS structure that is discussed in the text. (b) Viisum and GeoMx visualisations of L4 colored by pathology label or fluorescence correspondingly. Highlights for immune cell aggregation in Visium, and ROI/AOI types on GeoMx. Expression of Immune marker genes for regular and enhanced-by-BayesSpace data for Visium, and zoom-in regions for GeoMx with heatmaps showing separation of signal into the AOI-s. Black arrows point to the *Other* AOI, where the B-cell signal is expected to appear. Images above are representative of 1 sample. (c) GeoMx AOI-s from L4 colored by segment type, or B-cell, T-cell and Macrophage deconvolution fractions. (d) UMAP of Chromium B3 tumor nuclei with clusters gained by unsupervised clustering, annotated into Tu_B3_PLA2G2A and Tu_B3_NPPC malignant cell substates with marker genes PLA2G2A and NPCC expression on UMAP. (e) Spatial localisation of the two tumor subclusters to Areas A and B on patient B3 Visium by deconvolution cell type fraction. (f) DE of the two tumor substates (Wilcoxon's rank sum test, two-sided, multiple comparisons corrected with default Bonferroni method, adjusted p value < 0.05, log fold change > 1). Sample L1 - GeoMx: 25 AOIs. Sample L4 - Visium: 2,750 spots; GeoMx: 26 AOIs. Sample B3 - Chromium: 3,477 cells. Source data are provided as a Source Data file.

File Name: Supplementary Figure 10

Description: SpotClean and SCTransform integration on Visium reduce patient-specific batch-effect. UMAP plots on Breast (a, d, g, j), Lung (b, e, h, k) and DLBCL (c, f, i, l) patients on log normalized (a-f) and post-SCTransform (g-l) data, with (d-f, j-l) or without (a-c, g-i) SpotClean. Every dot represents a spot, colored by sample ID. Visium - Breast: 5 samples/10,086 spots; Lung: 5 samples/7,764 spots; DLBCL: 6 samples/18,580 spots. Source data are provided as a Source Data file.

File Name: Supplementary Figure 11

Description: SpotClean and SCTransform integration on Visium retain meaningful biology.

UMAP plots on Breast (a, b), Lung (c, d) and DLBCL (e, f) patients on post-SCTransform and post-SpotClean data. Every dot represents a spot, colored by pathology label (a, c, e) and deconvolution majority vote (b, d, f). Visium - Breast: 5 samples/10,086 spots; Lung: 5 samples/7,764 spots; DLBCL: 6 samples/18,580 spots. Source data are provided as a Source Data file.

File Name: Supplementary Figure 12

Description: Pre- and post batch correction TSNE plots on GeoMx data. TSNE plots on Breast (a-f), Lung (g-l) and DLBCL (m-r) patients on pre-batch-corrected (a-c, g-i, m-o) and post-normalisation (d-f, j-l, p-r) data. Every dot represents an AOI, colored by either Slide ID (a, d, g, j, m, p), Sample ID (b, e, h, k, n, q) or AOI label (c, f, i, l, o, r). Batch effect in lung samples is explained in g and h. GeoMx - Breast: 5 samples/112 AOIs; Lung: 5 samples/118 AOIs; DLBCL: 6 samples/136 AOIs. Source data are provided as a Source Data file.

File Name: Supplementary Figure 13

Description: Annotation of DLBCL Visium spots into regions. (a) UMAP of integrated DLBCL Visium samples, colored by Seurat cluster, with final annotations into regions. (b) Heatmap showing proportion of each cluster that belongs to spots from which patient. (c) Weighted counts in each sample by the inverse of its sample size. Heatmap showing proportion of each cluster that belongs to spots of which pathology annotation class. (d) Clustered dot plot showing canonical markers of known healthy cell types and gene expression level across all clusters, highlighting the assigned clusters to regions. (e) Aggregated patient-specific tumor marker (based on patient-specific malignant cell markers in Chromium in Supplementary Figure 4b-d) gene expression on UMAP of integrated DLBCL Visium samples. DLBCL: 6 samples/18,580 spots. Source data are provided as a Source Data file.

File Name: Supplementary Figure 14

Description: Ranking of drug target genes in DLBCL. Per-donor scores as -log10 p-values are shown with color-scale for every donor in every method Chromium, GeoMx and Visium for all the genes identified for Figure 6. Fold-changes are shown with the size of the symbol. Ranking of each gene within a donor is shown in either the top 100 genes by p-value by a square, or lower values by a circle. Source data are provided as Supplementary Data 9.

File Name: Supplementary Figure 15

Description: Chromium informed data-driven Visium and GeoMx inter-patient drug discovery. Deconvolution fractions in DLBCL for each targeted cell type in the corresponding majority voted cell type category in Visium (a) and for each targeted cell type in the corresponding AOI labeled segments in GeoMx (c). Higher purity is achieved by restricting the analysis to spots where the deconvolution majority cell type label's fraction is > 50% in Visium (b) and by filtering for segments where the AOI label and the deconvolution majority vote are in consensus in GeoMx (d). (e) Expression of drug targets as in Figure 6 with improved purity spots and AOI-s in Visium and GeoMx. Source data are provided as a Source Data file.

File Name: Supplementary Data 1

Description: Patient overview. A table with patient ID, Sample type, Cancer type, Tissue, Histology type, histology specification for Invasive lobular carcinoma, Immune histology, DV200 (%) as RNA quality value, Block age in months. NOS - Not Otherwise Specified. *Patient had a replica sample for GeoMx and Visium, **Patient had a replica sample for Visium, ***Patient had a replica sample for GeoMx.

File Name: Supplementary Data 2

Description: Single-cell samples. Information about single-cell samples: Sample ID, Patient ID, Sample repeating info, Cancer type, Chromium pool ID, Number and thickness of FFPE curls (micrometers), Pass/Fail status.

File Name: Supplementary Data 3

Description: GeoMx samples. Information about GeoMx samples: GeoMx slide ID, GeoMx run date, Patient ID, Section ID, Section letter on slide (A - closest to the label, C - furthest from the label if not otherwise specified), Comment on Pass/Fail status, FFPE section thickness, Staining mix with morphology marker information, Number of AOI-s collected. Calculated for each indication in μm^2: Minimum AOI area, Maximum AOI area, Mean AOI area, SD AOI area. AOI - area of illumination. SD - standard deviation.

File Name: Supplementary Data 4a

Description: DLBCL Lab Worksheet. Information about GeoMx AOI-s in DLBCL. Sample ID as AOI unique ID used in sequencing files, GeoMx slide name, GeoMx DSP scan name, transcriptome panel, Region of Interest ID (roi), Segment type based on morphology markers, Area of Illumination type (aoi), AOI area size in μm^2, Patient ID, Manually curated Cell fraction based on immunofluorescence image of morphology markers and the GeoMx DSP mask, AOI label with Manual curation, final AOI label with categories.

File Name: Supplementary Data 4b

Description: Breast Lab Worksheet. Information about GeoMx AOI-s in Breast as in DLBCL lab worksheet. Additional values: ROI name, ROI type, ROI_category, AOI label with location, Indication, Patient ID, Section ID, Unique ID human readable (hr), Comment, Tags, number of nuclei in AOI, ROI Coordinate X, ROI Coordinate Y, Scan Date.

File Name: Supplementary Data 4c

Description: Lung Lab Worksheet. Information about GeoMx AOI-s in Lung. All the same values as for Breast.

File Name: Supplementary Data 5

Description: Pathology annotation distribution. Pathology annotations, their grouping categories for Figure 2, suggested colors, and number of spots in each sample with the annotation.

File Name: Supplementary Data 6

Description: Single-cell annotations in DLBCL. Single-cell annotations at different levels, suggested colors and ontology terms. Level4 annotations, Harmonised Level 4 annotations across lung, breast and DLBCL, Harmonized colors suggestions, cell_type_ontology_term_id: CL, all other labels for Levels 3, 2.5, 2, 1.5 and 1. Level 1.5 generated as a mixture from Level 1 to 4 with cell type groups most similarly matching GeoMx segments. Matching equivalent GeoMX segment and colors.

File Name: Supplementary Data 7

Description: Single-cell annotations in Breast and Lung. Single-cell annotations at different levels,

suggested colors and ontology terms as in Supplementary Data 6.

File Name: Supplementary Data 8

Description: Gene-drug matches. Genes used in Manuscript Figures 5 and 6 as drug targets with associated drug molecule name and type, drug development status, drug and target's ChEMBL ID, and target's Ensembl ID. The information is pooled from the ChEMBL database [23].

File Name: Supplementary Data 9

Description: Drug target statistics. Fold changes, p-values and ranks for every donor and technology combination in malignant versus non-malignant compartment. Wilcoxon's rank sum test (two-sided, multiple comparisons corrected with default Bonferroni method) was used for Chromium and Visium, and moderated t-test (two-sided, multiple comparisons corrected with default Benjamini-Hochberg method, adjusted p value < 0.05, log fold change > 1) was used for GeoMx to find differentially expressed genes between clusters of interest.
